# Association of Fractional Anisotropy in White Matter Bundles with Plasma Biomarkers of Neurodegeneration and Glial Reactivity in Individuals with Cognitive Complaints without dementia

**DOI:** 10.21203/rs.3.rs-8621977/v1

**Published:** 2026-01-19

**Authors:** Patricio F. Riquelme, Isaias Mery, Sebastian Aguilera, Fernando Henriquez, Vicente Medel, Pamela C L. Ferreira, Bruna Bellaver, Thomas Karikari, Tharick A. Pascoal, Daniela Thumala, Cecilia Okuma, Patricia Lillo, J. Cesar Cardenas, Christian González-Billault, Felipe A. Court, Claudia Duran-Aniotz, Pamela Guevara, Andrea Slachevsky

**Affiliations:** Universidad de Chile; Universidad de Chile; Universidad de Chile; Universidad de Chile; Pontificia Universidad de Chile; University of Pittsburgh; University of Pittsburgh; University of Pittsburgh; University of Pittsburgh; Universidad de Chile; Universidad de Chile; Universidad de Chile; Universidad Mayor; Universidad de Chile; Universidad Mayor; Universidad Adolfo Ibanez; Universidad de Concepción; Universidad de Chile

## Abstract

Age-related cognitive decline is preceded by subtle neurochemical and structural brain alterations that remain insufficiently characterized, particularly in individuals with cognitive complaints but without dementia. Circulating plasma biomarkers reflecting amyloid pathology, tau phosphorylation, astroglial reactivity, and axonal injury have emerged as accessible indicators of early neurodegenerative processes; however, their relationship with early white matter microstructural changes remains unclear. In this study, we investigated associations between plasma Aβ42/Aβ40, phosphorylated tau 217 (p-tau217), glial fibrillary acidic protein (GFAP), and neurofilament light chain (NfL), and white matter integrity assessed by fractional anisotropy (FA) derived from diffusion MRI.

We analyzed data from 135 community-dwelling older adults with cognitive complaints but without dementia, recruited through a population-based strategy. Diffusion MRI data were processed using deterministic and correlational tractography to identify white matter pathways in which FA was significantly associated with plasma biomarker levels, controlling for age and education. Anatomical labeling of implicated tracts was performed using probabilistic white matter atlases.

Higher plasma levels of p-tau217 and GFAP were consistently associated with reduced FA across multiple periventricular white matter tracts, including the corpus callosum, fornix, optic radiations, and posterior thalamic radiations—pathways known to be vulnerable in early cognitive impairment. NfL exhibited a similar but more spatially restricted pattern, primarily involving callosal fibers. In parallel, direct associations between plasma biomarker levels and increased FA were observed in limbic-related bundles such as the cingulum and fornix, suggesting regionally specific microstructural responses that may reflect early adaptive or remodeling processes.

Together, these findings demonstrate that plasma biomarkers of neurodegeneration and glial reactivity are closely linked to white matter microstructural alterations at pre-dementia stages. This study provides converging neurochemical and neuroanatomical evidence supporting the utility of blood-based biomarkers as indicators of early brain structural vulnerability and reinforces their translational relevance in the study of age-related neurodegenerative processes.

## Introduction

Age-related cognitive impairment and dementia represent rapidly growing public health challenges worldwide, driven by the accelerated aging of the global population (Fang et al., 2025). Neurocognitive disorders, including Alzheimer’s disease dementia (ADD), are characterized by a prolonged preclinical phase that may extend for 15–20 years prior to the onset of overt dementia (Scheltens et al., 2021). Identifying individuals during these early stages is critical, as it provides a window of opportunity for preventive strategies and disease-modifying interventions. Evidence from high-income countries (HICs) indicates that molecular and neuroimaging biomarkers can contribute substantially to the identification of individuals at risk during preclinical phases or those presenting cognitive complaints without dementia (Shaikh et al., 2025).

Within this population, neuroimaging markers such as cortical atrophy, white matter hyperintensities (WMH), and alterations in functional connectivity have been consistently reported, even in the absence of objective neuropsychological impairment, when compared with cognitively unimpaired (CU) individuals (Dhana et al., 2022; Pievani et al., 2024; Ribaldi et al., 2024). These findings suggest that neurobiological changes may precede measurable cognitive decline, highlighting the relevance of sensitive imaging biomarkers for early disease detection.

Among these markers, white matter microstructural integrity has emerged as a particularly relevant neuroimaging biomarker, as alterations in white matter are thought to occur at very early stages of cognitive impairment (Chao et al., 2022; Wang et al., 2012; Wei et al., 2021). Fractional anisotropy (FA), derived from diffusion tensor imaging (DTI), is widely used to estimate microstructural properties of white matter tracts (Meneguzzo et al., 2019). In individuals with mild cognitive impairment (MCI) and ADD, reduced FA in major tracts, including the corpus callosum, fornix, and parahippocampal cingulum, has been associated with cognitive dysfunction, myelin degeneration, and axonal damage (Bergamino et al., 2021; Kantarci, 2014). Importantly, these microstructural changes may precede macroscopic atrophy, positioning FA as a sensitive biomarker for early disease processes (Kantarci, 2014; Li et al., 2023; Teipel et al., 2014). Furthermore, FA alterations have been shown to correlate with amyloid and tau positron emission tomography (PET), currently considered gold-standard methods for characterizing Alzheimer’s pathology along the ADD continuum (Carlson et al., 2021; Sintini et al., 2019). However, PET imaging remains invasive, costly, and largely inaccessible in many low- and middle-income settings, particularly in the Global South, underscoring the need for more accessible biomarkers that reflect early structural brain changes.

In this context, plasma biomarkers have gained increasing attention as minimally invasive, cost-effective tools with high prognostic accuracy for detecting neurodegenerative processes, even at early stages (Pais et al., 2023; Palmqvist et al., 2020). Neurofilament light chain (NfL) is a well-validated marker of axonal injury, and elevated plasma levels have been shown to predict accelerated cognitive decline and increased brain atrophy in individuals with cognitive complaints (Bangen et al., 2021; Götze et al., 2024). Glial fibrillary acidic protein (GFAP), a marker of astrocytic activation, is associated with cerebral amyloid pathology, with elevated plasma levels observed even in individuals with subjective cognitive complaints (Chatterjee et al., 2021, 2022). Among tau biomarkers, phosphorylated tau at threonine 217 (p-tau217) has demonstrated particularly high specificity for ADD, accurately discriminating tau and amyloid pathology and differentiating ADD from other dementias well before the onset of clinical impairment (Giacomucci et al., 2025; Palmqvist et al., 2020). Finally, the plasma Aβ42/Aβ40 ratio serves as an indirect indicator of cerebral amyloid burden, with lower ratios reflecting increased amyloid deposition in the brain. In individuals with cognitive complaints, reductions in this ratio correlate with amyloid PET findings and improve diagnostic accuracy when combined with GFAP (Chatterjee et al., 2022; Kang et al., 2025).

Despite the growing evidence supporting the utility of plasma biomarkers for early ADD detection, their relationship with early structural markers of brain damage—particularly white matter microstructural integrity—remains insufficiently characterized in individuals with cognitive complaints without dementia. Clarifying these associations is essential to strengthen the biological validity of plasma biomarkers as surrogates of early neurodegenerative and glial-related structural changes in the aging brain.

In a previous study conducted by our group, we demonstrated that WMH volume was associated with cardiovascular risk factors, particularly diastolic blood pressure, but not with plasma biomarkers of neurodegeneration (Riquelme Contreras et al., 2025). As WMHs represent a relatively late and macroscopic manifestation of white matter damage, these findings suggest that vascular mechanisms may precede—or operate independently from—detectable neurodegenerative biomarker changes during early cognitive decline. Accordingly, the assessment of white matter microstructure using diffusion-derived metrics such as FA may capture subtler and earlier alterations that are not yet expressed as WMHs, thereby offering a more sensitive window into the initial neurobiological processes linking white matter vulnerability with plasma markers of neurodegeneration and glial reactivity.

Therefore, in the present study, we aimed to investigate the associations between plasma biomarkers of neurodegeneration and glial activation (NfL, GFAP, p-tau217, and Aβ42/Aβ40) and the integrity of major white matter fiber bundles, as measured by FA derived from DTI. This study was conducted within the framework of the *GERO cohort*, a unique community-based sample of Chilean older adults with low educational attainment and cognitive complaints, in whom extensive clinical, cognitive, neuroimaging, and biomarker assessments have been performed (Slachevsky et al., 2020). While this cohort parallels well-established community-based studies from North America and Europe, it provides novel and muchneeded evidence from a Latin American context, contributing to a more globally representative understanding of early neurobiological changes associated with age-related cognitive decline.

## Material and methods

### Participants

We included 135 individuals (108 females) selected from a Chilean community-based cohort of individuals with cognitive complaints without dementia (GERO Cohort) (Slachevsky et al., 2020). In brief, we employed a door-to-door recruitment strategy to identify and enroll community-dwelling adults aged 70 years and older from the general population. Household visits were conducted across three districts of Santiago de Chile and selected for their socioeconomic diversity to ensure representativeness. Participants were included if they reported cognitive complaints by themselves or through a reliable proxy; they scored 0.5 or less on the Clinical Dementia Rating Scale (CDR), lived in the community, and had a knowledgeable informant. For self-reported cognitive complaints, the questionnaire included 11 items from Rabin et al. (2015) and additional questions developed and preliminarily validated by our group. Proxy-reported cognitive complaints were assessed using the AD8 questionnaire and other items validated by our group (Galvin et al., 2005; Molina-Donoso et al., 2023; Muñoz et al., 2010; Parrao et al., 2018; Rabin et al., 2015; Slachevsky et al., 2017). Participants were included if they responded positively to a predefined set of questions (see Supplementary Material). We excluded individuals with dementia, as identified by screening tests that documented both cognitive impairment (scores < 21 on the MiniMental State Examination, MMSE) and functional limitation (scores > 2 on the Pfeffer Functional Activities Questionnaire, PFAQ) (Arevalo-Rodriguez et.al., 2015; Pfeffer, R et.al., 1982). Participants were also excluded from the study if they had a history of institutionalization, illiteracy, visual and auditory deficit impeding cognitive evaluation and/or the capacity to maintain an independent living, severe mobility impairment, severe psychiatric or neurological disorders, or a lethal disease with a prognosis of less than one year of survival. Additionally, a trained neurologist evaluated all participants to verify that they met the inclusion criteria.

In addition, all selected individuals had the following available evaluation data: diffusion magnetic resonance imaging (dMRI) available in the database, and the neurodegeneration blood biomarkers Aβ42, Aβ40, obtaining Aβ42/Aβ40 ratio derived from these markers, and p-tau217. Based on evidence demonstrating the efficacy of p-tau217 and Aβ42/Aβ40 ratio in detecting amyloid pathology (Ashton et al., 2024; Mielke et al., 2022; Palmqvist et al., 2024), we used both plasma biomarkers as a measurement of neurodegeneration. Regarding glial reactivity markers, glial fibrillary acidic protein (GFAP) and neurofilament light chain protein (NfL) were selected because of their reported performance in detecting neuroinflammation and their correlation with neurodegeneration, amyloid status, and preclinical stages of dementia (Chatterjee et al., 2023; Ingannato et al., 2024; Pereira et al., 2021).

More details about the recruitment and a complete description of the study protocol are available in (Slachevsky et al., 2020). This published study protocol details the sample size calculation and justification.

The GERO project was approved by the Scientific Ethics Committee of the Eastern Metropolitan Health Service of Santiago, Chile. All participants provided written informed consent according to the principles expressed in the Declaration of Helsinki, as approved by the same ethics committee. The study protocol is registered at ClinicalTrials.gov (NCT04265482), and recruitment was realized between May 2017 and July 2021.

### Blood samples acquisition and processing

For neurodegeneration and glial reactivity biomarker assessment, blood samples were collected in the subject’s home through a vacuum system with venous access using 21 G or 23 G needles, depending on the subject’s venous caliber. Samples were divided into aliquots and frozen at −80°C. Samples were shipped to the University of Pittsburgh (Pennsylvania, USA), with continuous temperature monitoring to maintain the cold chain throughout transport until laboratory processing. Plasma biomarkers Aβ42, Aβ40, pTau217, GFAP, and NfL were quantified using a Neurology 4-Plex E (#103670) commercial assay kit (Quanterix, Billerica, MA, USA).

### MRI acquisition and processing

Diffusion MRI images were acquired using a 3T Skyra scanner (Siemens) at the Neurosurgery Institute Dr. Alfonso Asenjo (Santiago, Chile), using the following acquisition parameters: repetition time (TR) = 10,000 ms, echo time (TE) = 92 ms, field of view (FoV) = 240 mm, voxel size: 2.0 × 2.0 × 2.0 mm, slice thickness: = 2 mm, b-value = 1000 s/mm^2^, and 30 diffusion-encoding directions. To obtain DTI metrics, images were converted into NIFTI format (.nii) and analyzed using DSI Studio (https://dsi-studio.labsolver.org/). Preprocessing considered the diffusion gradient intensity (.bval) and the diffusion direction vectors (.bvec) to generate .src files, followed by visual quality control to ensure the integrity of the images (Yeh, Zaydan, et al., 2019). Additionally, a brain mask was generated to filter the background and improve reconstruction efficiency. Next, eddy current and head-eye motion correction were applied, followed by computation of the diffusion local model using q-space diffeomorphic reconstruction (QSDR). QSDR reconstruction parameters included an ODF sampling count of 1500, a diffusion sampling length ratio of 1.25, and normalization to the ICBM-152 template (Yeh & Tseng, 2011). A .fib file resulted from this preprocessing, containing the orientation information of white matter fibers, which served as the basis for deriving diffusion-based metrics such as FA. Finally, a connectometry database was created for FA, using the .fib files from all participants (Yeh et al., 2016).

Correlational tractography (Yeh et al., 2021) was then performed to identify the white matter pathways that have FA significantly correlated with each plasma biomarker (Aβ42/Aβ40, p-tau217, GFAP, and NfL). A nonparametric Spearman partial correlation was used to derive the correlation, and the effects of age and years of formal education were removed using a multiple regression model. The statistical significance of the correlation was examined using a permutation test. A T-score threshold of 2.5 was assigned in the fiber tracking algorithm (Yeh et al., 2013). A whole-brain seeding region was employed (MNI coordinates: 39,49,29). The resulting tracts were filtered using a topology-informed pruning (Yeh, Panesar, et al., 2019) with 16 iterations. A minimum length threshold of 15 voxels was used to select tracks. To estimate the false discovery rate (FDR), a total of 4000 randomized permutations were applied to the group labels to obtain the null distribution of the track length (FDR < 0.05).

Finally, tracts showing significant correlations were anatomically identified using probabilistic maps based on the HCP-1065 white matter tract atlas (Yeh, 2022), which includes 64 major white matter pathways. This anatomical identification step was performed using Python (version 3.10.14).

## Results

### Demographics, clinical and biomarkers

The average age of the participants was 76.09 years (SD = 4.93), with an average formal education of 9.36 years (SD = 4.66). Further, the sample exhibited a mean CDR total score of 0.45 (SD = 0.44) and a mean MMSE of 27.09 (SD = 2.28). Together, these values confirm that our cohort is devoid of dementia and represents individuals within the spectrum of cognitive complaints.

The average plasma concentration of Aβ42 and Aβ40 were 6.48 pg/mL (SD = 2.08) and 108.96 pg/mL (SD = 24.29), respectively. For p-tau217, the mean value was 0.23 pg/mL (SD = 0.20), GFAP averaged 138.50 pg/mL (SD = 70.80), and NfL averaged 29.91 pg/mL (SD = 14.01).

Demographic, clinical, and summary statistics for all blood biomarkers are presented in Table 1.

### Associations of FA with Aβ42/Aβ40

**Figure 1:**
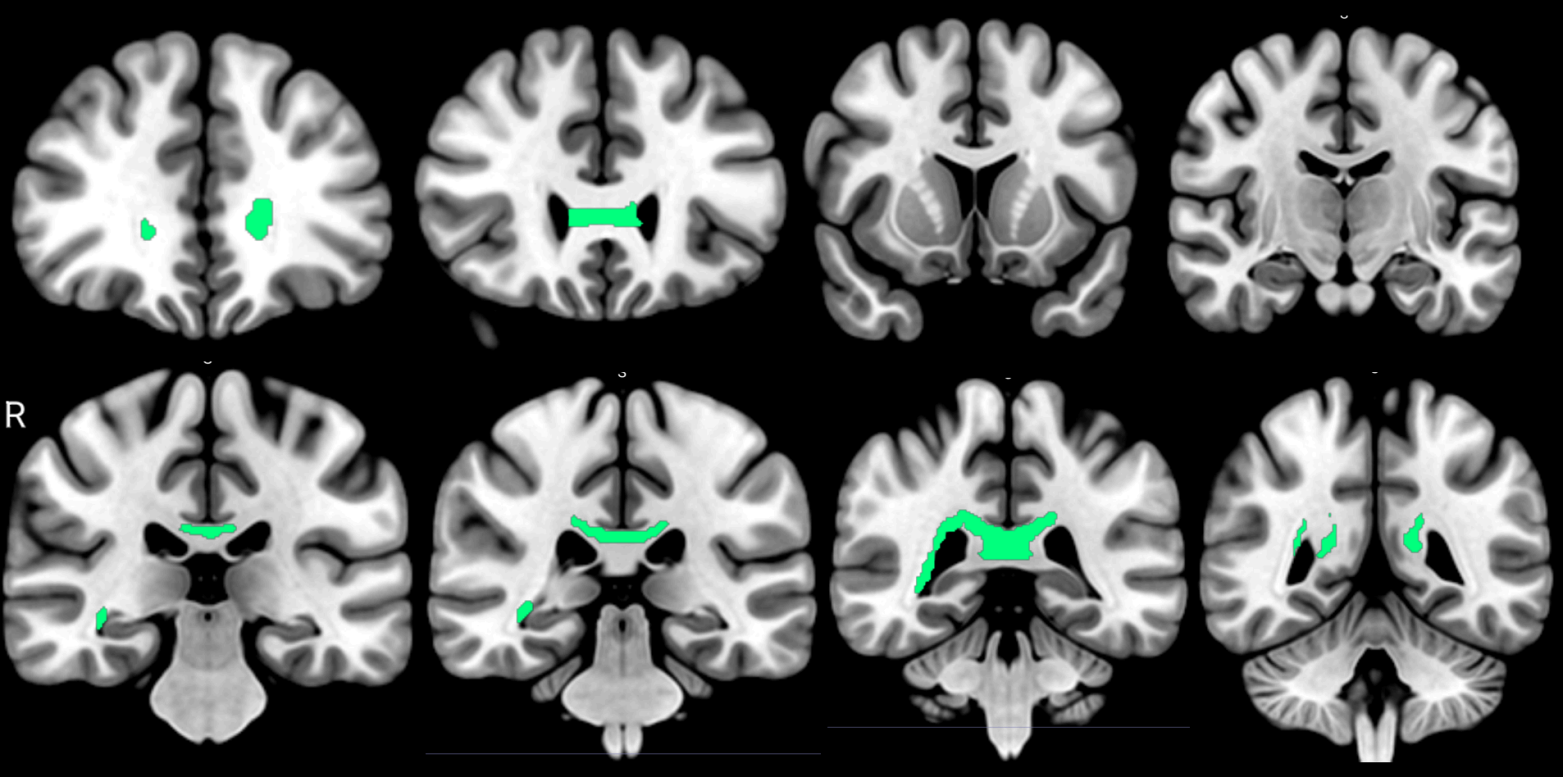
tracts with direct associations between FA and Aβ42/Aβ40 plasmatic levels (FDR < 0.05). It can be identified as the corpus callosum genu, corpus callosum forceps major, tapetum, and right optic radiation.

Correlational tractography to explore direct associations between Aβ42/Aβ40 plasmatic levels showed a special commitment to periventricular fibers from: corpus callosum tapetum (percent: 4.35%), right optic radiation (percent: 1.96%), right corticopontine tract occipital (percent: 1.79%), and right thalamic radiation posterior (percent: 1.72%). The total of tracts identified are shown in Table 2, with a graphical representation exposed in Fig. 1.

Regarding the inverse associations between FA and Aβ42/Aβ40 plasma levels, the tracts with a higher number of fibers in relation were both the fornix, with 8.46% and 6.97% of the total fibers compromised in the right and left, respectively (see supplementary material).

### Associations of FA with p-tau217

**Figure 2:**
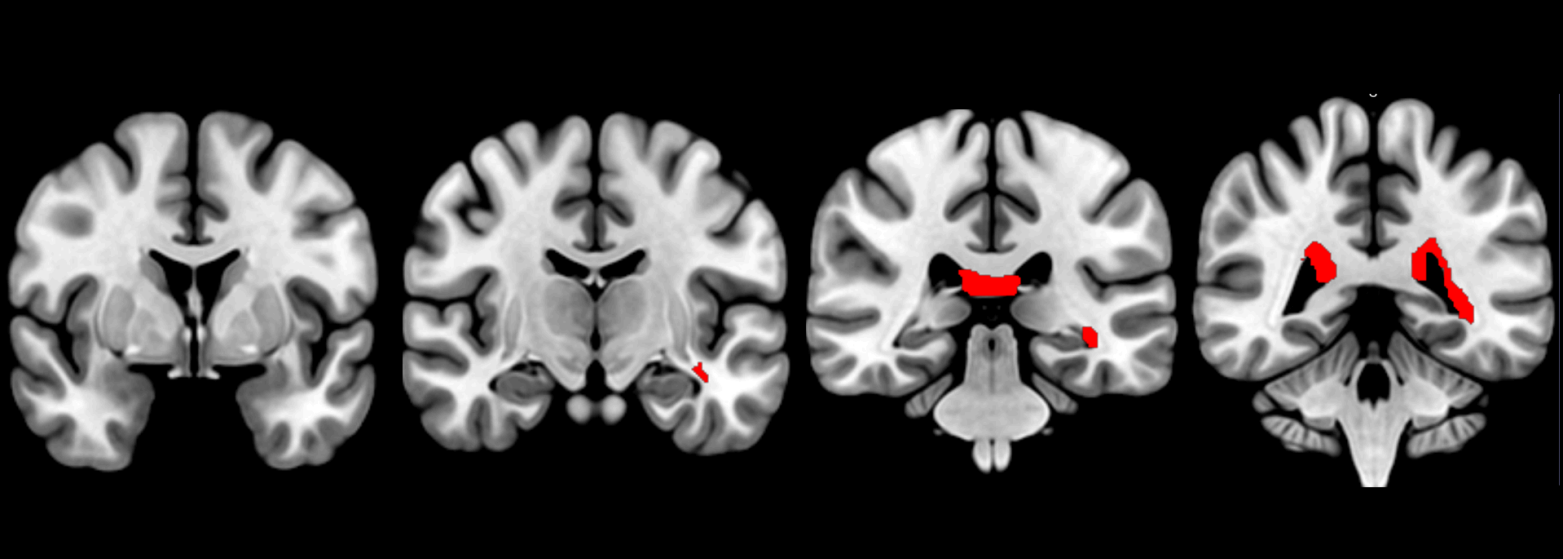
tracts with inverse associations between FA and p-tau217 plasmatic levels (FDR < 0.05). It can be identified tapetum and optic radiation.

Inverse associations between p-tau217 and FA affected, mainly, bundles from posterior periventricular white matter, highlighting the associations found in corpus callosum tapetum (percent: 8.86), right fornix (percent: 3.38%), and left optic radiation (percent: 3.29%). The total number of anatomically identified bundles is shown in Table 3. An anatomical map of these associations is represented in Fig. 2.

**Figure 3:**
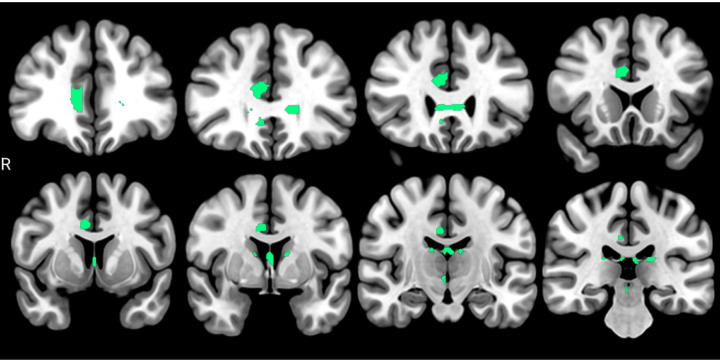
tracts with direct associations between FA and p-tau217 plasmatic levels (FDR < 0.05). It can be identified in the right cingulum, corpus callosum genu, and both fornix.

Regarding direct associations, bundles from the limbic circuit exhibited a higher number of fibers with associations. Within the bundles showing these associations are the right cingulum frontal parahippocampal, parietal, and paraolfactory, both fornix, and right superior longitudinal fasciculus. The quantification and proportion of identified voxels are reported in Table 4, with an anatomical representation shown in Fig. 3.

### Associations of FA with GFAP

**Figure 4:**
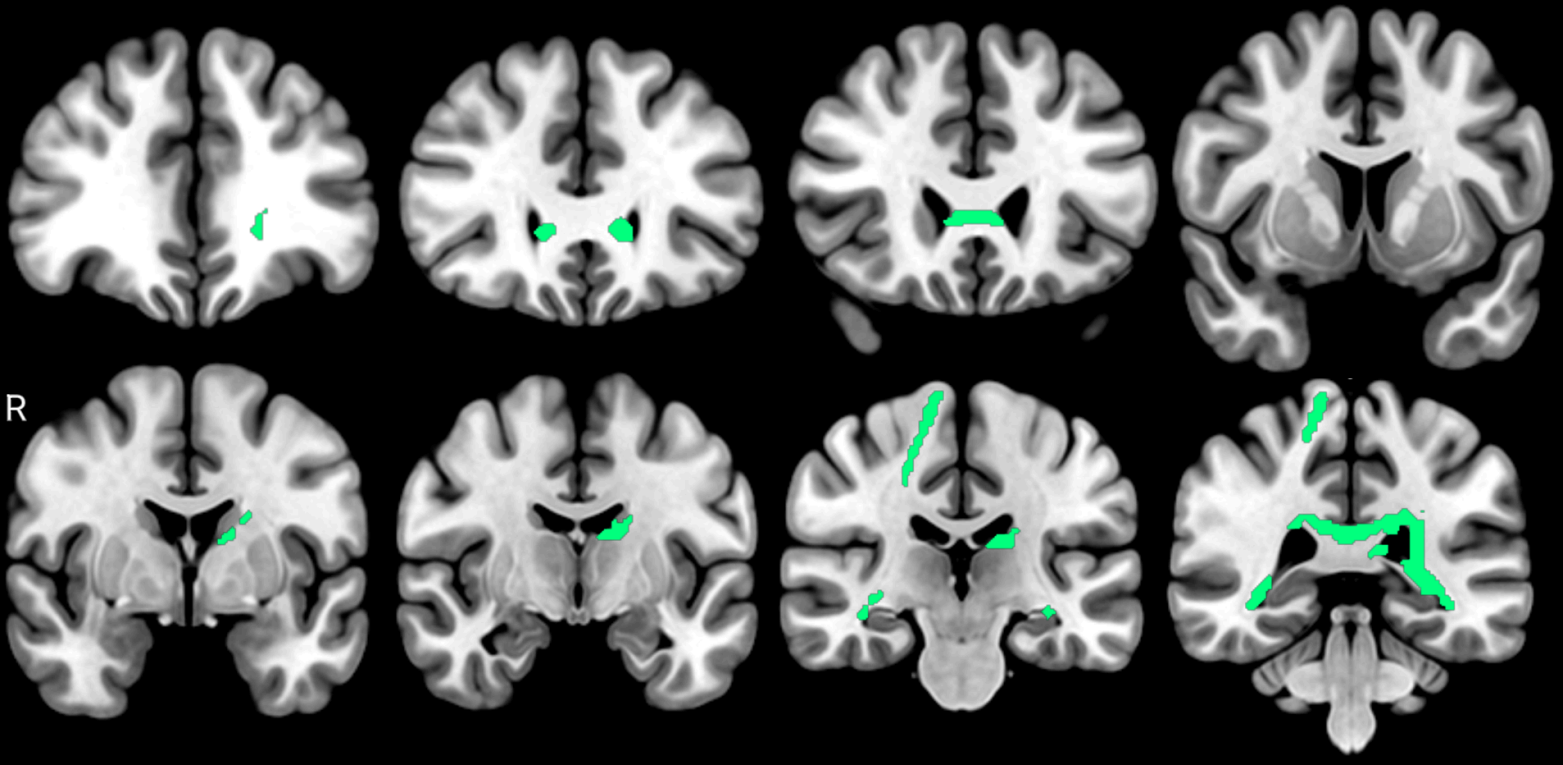
tracts with inverse associations between FA and GFAP plasmatic levels (FDR < 0.05). It can be identified corpus callosum genu, left thalamic radiation, corpus callosum tapetum, and optic tracts.

When calculating inverse associations between GFAP and FA, we found the highest number of bundles with relationships, with a strong compromise of posterior periventricular white matter. The corpus callosum tapetum had the highest percentage of fibers with association (12.26%), followed by the left fornix, and the right and left optic radiations. The bundles with the highest percentage of fibers with associations are shown in Table 5. An anatomical representation is exposed in Fig. 4.

Regarding direct associations, the cingulum and tapetum showed the highest associations. The complete report of them, and their anatomical localization, is presented in the supplementary material.

### Associations of FA with NfL

**Figure 5:**
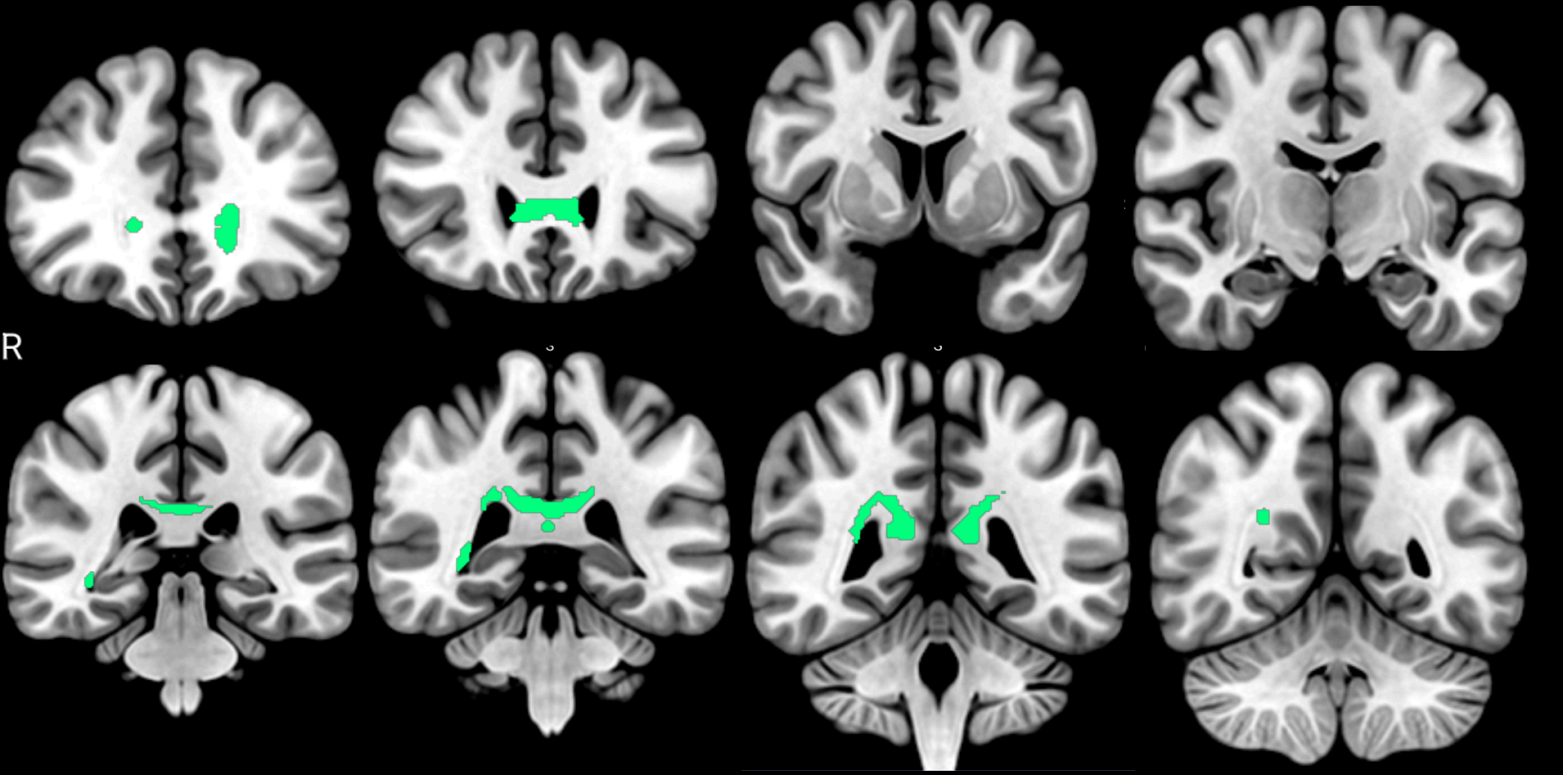
tracts with inverse associations between FA and NfL plasmatic levels (FDR < 0.05). It can be identified corpus callosum genu, thalamic radiation, corpus callosum tapetum, and right optic tract.

Inverse associations with NfL were marked for a compromise of the corpus callosum. Right thalamic radiation was also identified, but with a significantly lower number of voxels involved. The quantification of these associations is reported in Table 6, and their anatomical representation is shown in Fig. 5.

Finally, direct associations between NfL and FA were found in limbic circuit bundles, encompassing bilateral cingulum, fornix, and corpus callosum tapetum. The complete report of their quantification and anatomical representation is exposed in the supplementary material.

## Discussion

In this study, we examined the association between plasma biomarkers reflecting core neurobiological processes of neurodegeneration and glial reactivity; Aβ42/Aβ40, p-tau217, GFAP, and NfL, and white matter microstructural integrity, quantified by FA derived from deterministic tractography, in a community-dwelling older adults with cognitive complaints but without dementia. Our main findings indicate robust associations between plasma biomarkers and FA across multiple large-scale white matter fiber bundles. Specifically, higher plasma concentrations of p-tau217 and GFAP were predominantly associated with reduced FA in several periventricular and long-range tracts, while regionally selective positive associations were observed within limbic-related pathways. Overall, these findings suggest that early molecular alterations linked to amyloid–tau pathology, astroglial activation, and axonal injury are accompanied by tract-specific white matter microstructural changes at the earliest stages of the cognitive impairment continuum.

The white matter bundles most consistently involved in these associations included the corpus callosum, fornix, optic radiations, and cingulum, tracts known to be particularly vulnerable during early cognitive decline. These fiber bundles have been repeatedly implicated in individuals presenting subjective or objective cognitive complaints and have been associated with early alterations in memory, executive function, and cognitive integration (Griffanti et al., 2018; Sanderson-Cimino et al., 2021). Moreover, these tracts are anatomically positioned in periventricular and limbic regions that are especially susceptible to metabolic, vascular, and neurodegenerative insults, making them plausible early targets of pathological processes preceding overt dementia, and have been reported to be associated with early amyloid- and tau-related neurochemical disturbances (Brown et al., 2019; Kantarci et al., 2017), as well as astroglial activation (Bettcher et al., 2024).

Although reference intervals for plasma biomarkers of neurodegeneration have not yet been established in this population, some evidence suggests that reference intervals for Aβ42 and Aβ40 are between 2.72 − 11.09 pg/mL and 61.4–303.9 pg/mL, respectively (Chen et al., 2023). The same case is for p-tau217, where some evidence has established levels > 0.46 pg/mL as a cut-off for discriminating Aβ status (Olvera-Rojas et al., 2024). Based on these thresholds, we could confirm that our participants would mainly present a negative Aβ status. Regarding glial reactivity biomarkers, emerging evidence proposes cut-offs of 127 pg/mL for GFAP and 15.68 pg/mL for NfL to differentiate controls vs preADD (Doecke et al., 2025). However, interpretation should be made with caution, in the context of the neurodegeneration marker levels in our sample, as well as p-tau217 levels and the clinical characteristics of the cohort.

Regarding the associations between FA and the plasma biomarkers examined, GFAP and p-tau217 showed the greatest number and strength of associations with white matter FA. This finding is consistent with growing evidence positioning these biomarkers among the most sensitive indicators of amyloid pathology and risk of progression to ADD (Capuano et al., 2025; Hansen & Wiltfang, 2025; Imperiale et al., 2025; Kleiman et al., 2025; Sakurai et al., 2025). As a marker of astrocytic activation, GFAP reflects neuroinflammatory and reactive glial processes that may directly influence white matter integrity through myelin remodeling, metabolic support dysfunction, or neurovascular coupling alterations. Consistent with this interpretation, plasma GFAP has been shown to effectively differentiate ADD dementia from other neurodegenerative diseases and to predict individual risk of ADD progression (Zheng et al., 2024). In parallel, plasma p-tau217 has demonstrated high specificity for ADD pathology and strong concordance with tau-PET findings, even in preclinical and prodromal stages (Kim et al., 2025). Similar elevations have been reported for other plasma p-tau isoforms (p-tau181, p-tau217, and p-tau231) in ADD and MCI, broadly paralleling tau-PET findings. Taken together, these results support the integration of blood-based tau assays as surrogate measures for imaging biomarkers, helping bridge the gap between research tools and routine clinical practice.

In contrast, NfL showed fewer and more regionally restricted associations, consistent with its role as a more general marker of axonal injury that may become more prominent as neurodegeneration advances. The absence of robust associations with the plasma Aβ42/Aβ40 ratio may reflect the indirect nature of this biomarker and its stronger relationship with cortical amyloid burden rather than white matter microstructure per se (Mitolo et.al., 2024).

An important interpretative framework for our findings arises from emerging evidence indicating that white matter microstructural degeneration, reflected by decreased FA, systematically precedes macrostructural changes that ultimately manifest as WMH on FLAIR MRI. In particular, it has been reported that lower FA levels fully explain Aβ-related WMH burden and partially mediate age-related WMH accumulation, positioning FA as an upstream marker of white matter vulnerability (Sun et al., 2025). Within this context, our results suggest that plasma biomarkers, particularly p-tau217 and GFAP, may capture early microstructural white matter alterations that anticipate the development of overt WMH. These findings support a model in which molecular neurodegenerative and glial processes precede and possibly contribute to the later emergence of macroscopic white matter lesions.

When contrasted with our prior work, in which no associations were observed between plasma biomarkers and WMH volume, the present findings can be interpreted as reflecting differences in the spatial and biological scale of the measurements employed. Whereas WMHs represent a relatively late and coarse manifestation of white matter damage, diffusion-derived metrics such as FA are sensitive to subtle alterations in axonal organization and myelin integrity. Accordingly, our results support the hypothesis that, at the earliest stages of cognitive decline, white matter microdegeneration may be more closely linked to amyloid–tau pathology and astroglial reactivity than to vascular mechanisms detectable through WMH burden. Longitudinal analyses will be necessary to determine whether these microstructural changes evolve into overt WMHs and how plasma biomarkers track this progression over time.

Several limitations should be acknowledged. First, the sample exhibited an underrepresentation of men, a pattern commonly observed in aging research due to differential participation rates in health-related studies (Abdelnour et al., 2017; Forno et.al., 2025). Third, the lack of gold-standard biomarkers such as cerebrospinal fluid measures or amyloid PET—due to limited accessibility and invasiveness, particularly in Global South settings—precluded direct validation of amyloid and tau status (McGlinchey et al., 2024). Additionally, the absence of locally validated reference ranges for plasma biomarkers required reliance on externally published cut-off values (Chen et al., 2023; Olvera-Rojas et al., 2024). Finally, white matter bundle identification was based on probabilistic atlas-based labeling of reconstructed tracts, an approach that, while widely used, introduces uncertainty related to inter-individual anatomical variability and partial overlap between adjacent fiber pathways.

In conclusion, this study demonstrates that plasma biomarkers of neurodegeneration and glial reactivity, particularly p-tau217 and GFAP, are associated with alterations in white matter microstructural integrity in community-dwelling older adults with cognitive complaints but without dementia. By linking accessible blood-based biomarkers with diffusion-derived measures of large-scale white matter fiber integrity, our findings extend current knowledge of the neurobiological substrates underlying early cognitive decline. These results support the view that microstructural white matter alterations are detectable at the earliest stages of the dementia continuum and reinforce the potential clinical utility of plasma biomarkers as indicators of underlying brain structural vulnerability, especially in resource-limited settings. Longitudinal, multimodal studies will be essential to elucidate the temporal dynamics of these associations and to determine their value for early risk stratification and intervention in age-related neurodegenerative processes. Further, to explore white matter microstructural associations using a clinical ADD group could help for a better contextualization of the observed associations across the dementia continuum.

## Figures and Tables

**Table 1:** Description of demographic, clinical data, and plasma biomarker levels in the sample.

Variable	Mean (Median)	SD	Min	Max
Age (years)	76.096 (75)	4.93	69	92
Education (years)	9.363 (8)	4.66	0	24
MMSE Total Score	27.090 (28)	2.28	20	30
CDR Total Score	0.450 (0.5)	0.16	0	0.5
PFAQ Total Score	0.766 (0)	1.28	0	8
Aβ42 (pg/ml)	64.817 (64.04)	17.73	13.766	118.103
Aβ40 (pg/ml)	108.968 (105.87)	21.29	58.749	196.607
Aβ42/Aβ40 ratio	0.050 (0.060)	0.01	0.01	0.09
p-tau217 (pg/ml)	0.239 (0.167)	0.20	0.025	17.288
NfL (pg/ml)	29.915 (27.88)	14.01	10.131	93.606
GFAP (pg/ml)	138.507 (124.79)	70.80	36.982	503.720

**Table 2:** results of the anatomical bundle identification regarding direct associations between FA and Aβ42/Aβ40 plasmatic levels (FDR < 0.05).

Bundle	Bundle voxel n	Bundle voxel int	%
Corpus callosum tapetum	44230	1925	4.352
Right optic radiation	21010	413	1.965
Right corticopontine tract occipital	26345	473	1.795
Right thalamic radiation posterior	35747	615	1.72
Corpus callosum forceps major	117410	1874	1.596
Right corticostriatal tract posterior	51564	585	1.134

Bundle voxel n: number of voxels for the bundle according to the HCP 1065 white matter atlas; Bundle voxel int: number of fibers with intersection between the atlas and fibers correlated; %: percent of the bundle compromised by the association.

**Table 3:** Results of the anatomical bundle identification regarding inverse associations between FA and p-tau217 plasmatic levels (FDR < 0.05).

Bundle	Bundle voxel n	Bundle voxel int	%
Corpus callosum tapetum	44230	3919	8.860
Right fornix	2896	98	3.383
Left optic radiation	23529	775	3.293
Corpus callosum forceps major	117410	2287	1.947
Left fornix	2397	39	1.627
Left thalamic radiation posterior	38864	462	1.188
Right thalamic radiation posterior	35547	374	1.046
Left corticostriatal tract posterior	51578	534	1.035
Left corticopontine tract occipital	26840	273	1.017
Right optic radiation	21010	211	1.004

see Table 2.

**Table 4:** results of the anatomical bundle identification regarding direct associations between FA and p-tau217 plasmatic levels (FDR < 0.05).

Bundle	Bundle voxel n	Bundle voxel int	%
Right cingulum frontal parahipp	15661	2058	13.140
Right cingulum paraolfactory	12597	1631	12.947
Left fornix	2397	185	7.717
Right cingulum frontal parietal	30204	2155	7.134
Right fornix	2896	204	7.044
Right superior longitudinal fasc	24418	616	2.522

see Table 2.

**Table 5:** results of the anatomical bundle identification regarding inverse associations between FA and GFAP plasmatic levels (FDR < 0.05).

Bundle	Bundle voxel n	Bundle voxel int	%
Corpus callosum tapetum	44230	5424	12.263
Left fornix	2397	124	5.173
Left thalamic radiation posterior	38864	1940	4.991
Left optic radiation	23529	791	3.361
Right optic radiation	21010	689	3.279
Corpus callosum forceps major	117410	3471	2.956

see Table 2. See supplementary material for the complete report of the identified bundles.

**Table 6:** Results of the anatomical bundle identification regarding inverse associations between FA and NfL plasmatic levels (FDR < 0.05).

Bundle	Bundle voxel n	Bundle voxel int	%
Corpus callosum tapetum	44230	1788	4.042
Corpus callosum forceps major	117410	1669	1.421
Corpus callosum forceps minor	115178	1205	1.046
Right thalamic radiation posterior	35747	367	1.026

see Table 2. See supplementary material for the complete report of the identified bundles.
